# Long non-coding RNA terminal differentiation-induced non-coding RNA regulates cisplatin resistance of choroidal melanoma by positively modulating extracellular signal-regulated kinase 2 via sponging microRNA-19b-3p

**DOI:** 10.1080/21655979.2021.2014618

**Published:** 2022-01-22

**Authors:** Wei Li, Aiqin Nie, Longyu Jin, Yubo Cui, Ning Xie, Gaohua Liang

**Affiliations:** a Department of Ophthalmology, The Second Clinical Medical College of Jinan University, Shenzhen People's Hospital, Shenzhen City, Guangdong Province, 518000 P. R. China; b Department of Ophthalmology, The Affiliated Hospital of Youjiang Medical University for Nationlities, Guangxi, Baise, 533000, China

**Keywords:** TINCR, miR-19b-3p, ERK-2, choroidal melanoma, cisplatin

## Abstract

In the present study, we aimed to investigate the role of long non-coding RNA terminal differentiation-induced non-coding RNA (TINCR) in cisplatin (DDP) resistance of choroidal melanoma (CM) and the potential molecular mechanisms. CM and non-CM tissues were collected from 60 CM patients. DDP-resistant CM cells were obtained by selection with linearly increased DDP treatment. The expression levels of TINCR, microR-19b-3p (miR-19b-3p), and extracellular signal-regulated kinase 2 (ERK-2) were detected by quantitative real-time PCR. Cholecystokinin octapeptide (CCK-8) assay was utilized to detect chemosensitivity and cell viability. Flow cytometry analysis was performed to detect apoptotic cells. The protein levels of Bax, Bcl-2, cleaved-caspase-3, ERK-2, and nuclear factor-kappa B p65 were measured by Western blot. RNA immunoprecipitation (RIP) and dual-luciferase reporter assays were performed to determine the relationship among TINCR, miR-19b-3p, and ERK-2. The results showed that the levels of TINCR and ERK-2 were markedly increased in DDP-resistant CM tissues and cells, while miR-19b-3p level was significantly reduced. TINCR knockdown reduced DDP resistance and cell viability and promoted cell apoptosis, while TINCR overexpression exhibited opposite effects. TINCR and ERK-2 were direct targets of miR-19b-3p. Further experiments revealed that TINCR enhanced DDP resistance in CM cells by regulating the miR-19b-3p/ERK-2/NF-kb axis. Taken together, our study revealed a critical role of TINCR in regulating DDP resistance in CM and suggested that TINCR is a potential cisplatin-resistant CM therapeutic target.

## Introduction

Choroidal melanoma (CM) is a common malignant tumor of the eye and is prone to occur in middle-aged and elderly men. Its symptoms are more insidious and mainly manifested in vision decline and occlusion [1]. CM has a very high degree of malignancy and can infiltrate the surrounding tissues in a short period of time. CM can also metastasize early and distantly by blood route, mostly to the liver [2]. Studies have found that the average survival of CM patients with liver metastasis is only six months. The pathogenesis of CM is very complicated, involving various factors [3]. The main treatment of CM is enucleation, which significantly affects the quality of life of patients [4]. Currently, there is no effective treatment for CM patients with distant tissue metastasis [5]. Moreover, existing treatments have limited efficacy due to primary and secondary drug resistance. Studies have shown that most CM patients have primary or secondary tolerance with platinum-based chemotherapy [6]. Cisplatin (DDP), the most used platinum-based chemotherapy drug, can promote tumor cell apoptosis by inhibiting DNA replication. Cisplatin resistance is an important factor leading to treatment failure and affecting the survival and prognosis of CM patients. In order to better understand the causes of tumor cell proliferation and metastasis and to avoid programmed death of normal cells [7], it is critical to elucidate the mechanism of CM tolerance to platinum drugs and to find effective drug resistance markers.

Long non-coding RNAs (LncRNAs), a type of RNAs with lengths greater than 200 nucleotides, have no protein-coding region or have a region coding less than 100 amino acids [8]. LncRNAs are involved in gene regulation at epigenetics, transcriptional, and post-transcriptional levels [9–11]. In recent years, lncRNAs and DDP resistance have become hot research spots [12]. For example, lncRNA ENST00000457645 plays a negative role in cisplatin-resistant ovarian cancer cell survival and migration with underlying mechanisms related to regulating apoptotic proteins [13]. LncRNA terminal differentiation-induced non-coding RNA (TINCR) is a newly discovered tumor-associated lncRNA that regulates related bladder cancer biological processes [14]. It has been reported that TINCR promotes the resistance of breast cancer to cisplatin, but the effect of TINCR on CM cisplatin resistance remains unknown [15]. Therefore, it is important to explore the mechanism of TINCR in CM cell resistance to cisplatin.

LncRNAs can regulate mRNA expression or bind to microRNA (miRNA) to affect their downstream target gene expressions [16]. The abnormal expression of miRNAs has been found to be involved in the tumorigenesis and malignant process. Studies have identified abnormal expression profiles of miRNAs in many malignant tumors [17]. Many studies have focused on the roles of miRNAs in chemotherapy resistance of tumors, especially cisplatin resistance [18]. More and more miRNAs have been revealed to be related to cisplatin. For example, transfection of miR-30a-5p inhibitor or mimic could change melanoma cell sensitivity to cisplatin. MiR-30a-5p knockout affects melanoma cell drug resistance by targeting IGF1R gene [19]. MiR-19b-3p participates in certain tumor proliferation and drug resistance [20]. Studies have found that miR-19b-3p is involved in colon cancer proliferation and drug resistance by targeting mothers against decapentaplegic homolog 4 (SMAD4) [21]. Changes in miRNA expression result in abnormal expression of target genes, eventually changing tumor cell drug sensitivity [22]. ERK2 is an important member of MAPK family. Studies have shown that ERK2 signaling pathway is related to growth factors- and cytokines-regulated cell proliferation [23]. It has been found that cisplatin treatment could activate ERK2 in OVCAR-3/CDDP and OVCAR-3 cells and ERK2 activation in the nucleus could enhance cisplatin resistance [24]. Studies have found that chemotherapeutic drugs can induce expression of nuclear factor kappa B (NF-κB) and various inflammatory cytokines in different tumor cell lines and significantly reduce the apoptotic effect of chemotherapeutic drugs, suggesting that NF-κB mediates the inflammatory signaling pathway [25]. Our preliminary bioinformatic analysis has shown that TINCR and ERK2 are potential targets of miR-19b-3p. Hence, we hypothesized that TINCR might play a critical role in DDP resistance of CM by regulating the miR-19b-3p/ERK2 axis. In the present study, we aimed to investigate the role of TINCR in the DDP resistance of CM and its functions by regulating the miR-19b-3p/ERK2 axis.

## Methods

### Tissue samples

The tumor tissues and adjacent non-tumor tissues (2 mm distant from the main tumor) were collected from CM patients (n = 60) in Shenzhen People’s Hospital from 2015 to 2018. Patients were classified based on their DDP sensitivity or resistance, as reported previously [26]. Patients who were not resistant to DDP underwent primary CM surgery and received DDP chemotherapy. Patients who were not resistant to DDP did not undergo surgery. All patients signed the informed consent. This study was approved by the Ethics Committee of Shenzhen People’s Hospital (Approval Number: SPH623).

### Cell culture and transfection

CM cell line, OCM-1, was purchased from Shanghai Fudan Cell Resource Center (Shanghai, China). The OCM-1 cells were continuously exposed to gradually increased cisplatin concentrations (0, 2, 4, 6, 8, 10 μg/mL), and the surviving cells grew normally till the 12th passage. The DDP-resistant OCM-1 cell line, OCM-1/DDP, was cultured in DMEM containing 2 μg/mL DDP and 10% fetal bovine serum (FBS, Gibco, USA) to maintain drug resistance. All cells were maintained with 5% CO_2_ at 37°C. TINCR small interfering RNA (si-TINCR) and scramble control (si-NC), miR-19b-3p mimic, inhibitor and negative controls were obtained from GenePharma (Shanghai, China). PcDNA3.1 vector (ThermoFisher Scientific, Cleveland, OH, USA) was used to construct TINCR or ERK2-overexpression plasmid (pcDNA/TINCR or pcDNA/ERK2). For transfection, cells were transfected with 0.5 µg plasmids or 50 nM oligonucleotides using Lipofectamine 3000.

### Cholecystokinin octapeptide (CCK-8) assay

Cells were seeded into 96-well plates at a density of 3 × 10^3^/well with different DDP concentrations for 24 h. Then, CCK-8 assay was performed by adding 10 µL CCK-8 reagent (CCK-8 assay kit, Dojindo, Japan) into each well for another 2 h. Finally, the absorbance at 450 nm was measured using a microplate reader (Thermo Fisher Scientific, Cleveland, OH, USA).

### Quantitative real-time PCR (RT-qPCR)

Total RNAs were extracted using TRIzol reagent (Hualan, Shanghai, China). RNA quality and concentration were analyzed using NanoDrop 1000 (NanoDrop Technologies, Wilmington, USA). Then total RNAs were reversely transcribed into cDNA using qScript microRNA cDNA synthesis kit (Quantabio, Beverly, MA, USA), and qRT-PCR reactions were conducted on the 7500 real-time PCR system. GAPDH and U6 were selected as the endogenous controls. The relative levels of TINCR, miR-19b-3p, ERK2, and NF-κB were calculated using the 2^−ΔΔCt^ method [27]. The primer sequences are listed in Table 1.

**Table 1.** The primer sequences used in RT-qPCR

Gene name	Forward primers (5ʹ-3ʹ)	Reverse primers (5ʹ-3ʹ)
TINCR	CTAAATTACCTGGCCGCAG	CGCCTGAATTCCAAAGG
MiR-19b-3p	CTGGATGTGGAGCCATTGT	GTCCTTTCACCTGGGGCCGG
ERK-2	TGTGGTCCTCCCTCCTCS	CGCCTTCTCTCCGATGT
GAPDH	CGAGAGGATCCGCCGACAT	TTGTGCCATACAGCGTTGAC
U6	GACAGAATCGGTCTGTTGCAC	GATTACCCGTCCGCAATCGATC

### Western blot

Total proteins were extracted from tissues and cells using RIPA buffer and quantified using a BCA Protein Detection Kit (Beyotime, Beijing, China). A total of 0.5 μg total protein was separated on PAGE-SDS gels for 1.5 h and transferred onto PVDF membranes. The membranes were blocked in 5% skimmed milk for 4 h and incubated first with primary antibodies against ERK2 (1:1,000, Amyjet, Wuhan, China), NF-κB (1:1,000, Amyjet, Wuhan, China), GAPDH (1:1,000, Amyjet, Wuhan, China), and ERK (1:1,000, Amyjet, Wuhan, China) overnight and then with secondary antibody (1:1,000) for 1 h [28]. The signals were visualized using ECL substrate and analyzed using ImageJ.

### RNA immunoprecipitation (RIP)

RIP assay was conducted by RNA Immunoprecipitation Kit (Millipore, Bedford, MD, USA) following a previous protocol [29]. OCM-1/DDP cells were lysed, and the supernatants were incubated with protein A/G beads conjugated with antibodies that recognized immunoglobulin G (IgG) or argonaute-2 (Ago2). The relative RNA expression levels were assessed by RT-qPCR assay.

### Flow cytometry assay

After 48 h, DDP-resistant cells transfected with different vectors were harvested. Annexin V-FITC/PI kit (Solarbio, Beijing, China) was carried out to detect the apoptotic cells according to the manufacturer’s instructions.

### Dual-luciferase reporter assay

Dual-luciferase reporter assay was performed following a previous protocol [30]. The 3′-UTRs of TINCR and ERK-2 containing mutant (MUT) or wild-type (WT) miR-19b-3p binding sequence were cloned into psiCHECK™-2 luciferase plasmid. OCM-1/DDP cells were co-transfected with miR-19b-3p mimics (or NC) and TINCR-WT, TINCR-MUT, ERK-2-MUT or ERK-2-WT. The luciferase activity was detected by a dual luciferase assay kit (Promega, Madison, WA, USA) after transfection for 48 h.

### Statistical analyses

Data were analyzed by SPSS ver. 19.0 software (SPSS Inc., Chicago, IL, USA) and shown as mean ± standard deviation (mean ± SD). Differences among multiple groups were analyzed using one-way ANOVA. Pearson’s correlation analysis was used to evaluate the correlation among TINCR, miR-19b-3p, and ERK-2 expression in tissues. *P* < 0.05 indicated significant differences.

## Results

### TINCR is upregulated in DDP-resistant CM tissues and cells

**Figure 1.**
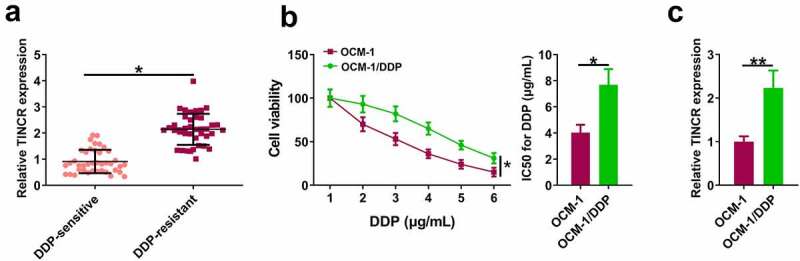
TINCR is upregulated in DDP-resistant CM. (a) TINCR expression in DDP-resistant or DDP-sensitive CM tissues was evaluated by RT-qPCR. (b) CCK-8 assay assessed DDP-resistant or DDP-sensitive CM viability and IC_50_. (c) INCR expression in DDP-resistant or DDP-sensitive CM cells was measured by RT-qPCR. *P < 0.05; **P < 0.01, n = 3.

We first explored TINCR expression level in DDP-resistant CM. RT-qPCR assay showed that TINCR level was significantly higher in DDP-resistant CM tissues than in DDP-sensitive CM tissues (Figure 1(a), p < 0.05). CCK8 assay revealed that DDP inhibited OCM-1 and OCM-1/DDP cell viability in a dose-dependent manner. However, OCM-1/DDP cells had higher viability than OCM-1 cells when exposed to the same DDP concentration, and OCM-1/DDP cells had higher IC_50_ than OCM-1 cells (Figure 1(b), p < 0.05). In addition, TINCR level was higher in OCM-1/DDP cells than in OCM-1 cells (Figure 1(c), p < 0.05).

### TINCR increased DDP resistance of CM cells

**Figure 2.**
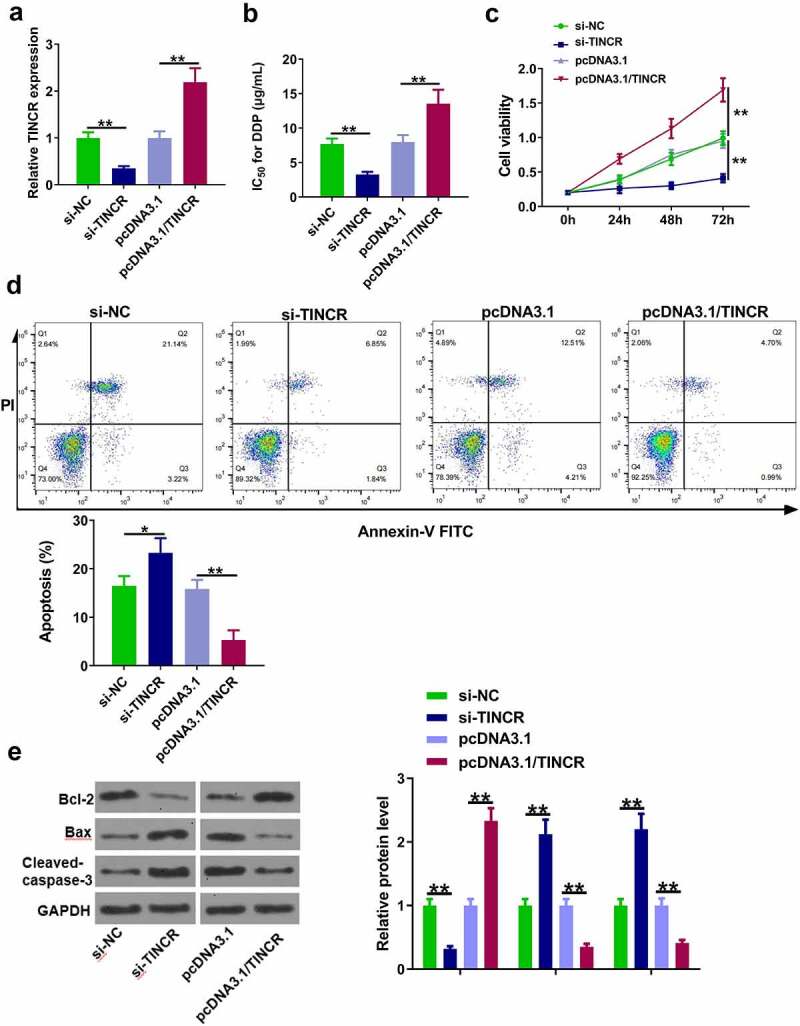
TINCR increases DDP resistance of CM cells. (a) RT-qPCR was used to determine TINCR expression in OCM-1/DDP cells after transfection. (b) IC_50_ values were measured by CCK-8 assay in OCM-1/DDP cells after transfection. (c, d) To detect OCM-1/DDP cell viability and apoptosis after transfection by CCK-8 and flow cytometry assays. (e) Bcl-2, Bax, and cleaved-caspase-3 expression were detected by Western blot analysis. *P < 0.05; **P < 0.01, n = 3.

Next, the role of TINCR in DPP-resistant CM cells was investigated. OCM-1/DDP cells were transfected with si-TINCR and pcDNA3.1/TINCR to inhibit and enhance TINCR expression, respectively (Figure 2(a), p < 0.05). The results showed that TINCR knockdown significantly reduced the IC_50_ value, while TINCR overexpression obviously increased the IC_50_ value (Figure 2(b), p < 0.05). CCK-8 assay showed that OCM-1/DDP cell viability was inhibited by TINCR knockdown and enhanced by TINCR overexpression (Figure 2(c), p < 0.05). Flow cytometry assay showed that apoptotic OCM-1/DDP cells were increased by TINCR knockdown and decreased by TINCR overexpression (Figure 2(d), p < 0.05). In addition, the protein levels of Bax, Bcl2, and cleaved-caspase-3 were also detected. The results showed that TINCR knockdown reduced Bcl-2 and increased Bax and cleaved-caspase-3, while TINCR overexpression exhibited opposite effects (Figure 2(e), p < 0.05). These data suggested that TINCR enhanced the DDP resistance of CM cells.

### TINCR directly bound to miR-19b-3p in DDP-resistant CM cells

**Figure 3.**
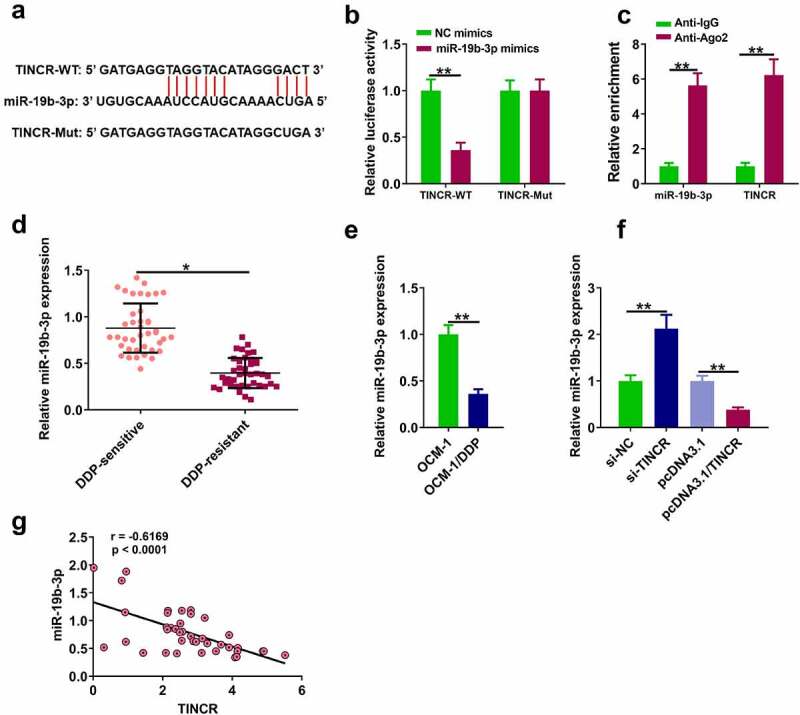
TINCR interacts with miR-19b-3p. (a) The interaction of TINCR and miR-19b-3p was detected by bioinformatics analysis. (b) Luciferase activity of TINCR-WT was detected by luciferase reporter assay. (c) RIP assay was used to detect the correlation between TINCR and miR-19b-3p. (d) MiR-19b-3p expression in DDP-resistant or DDP-sensitive CM tissues was measured by RT-qPCR. (e) MiR-19b-3p expression in DDP-resistant or DDP-sensitive CM cells was measured by RT-qPCR. (f) MiR-19b-3p expression in OCM-1/DDP cells after transfection was detected. (g) Correlation between TINCR and miR-19b-3p expression. **P < 0.01, *P < 0.05; n = 3.

The molecular mechanisms by which TINCR functioned in DP-resistant CM cells were explored. MiR-19b-3p was identified as a potential target for TINCR by Starbase ver. 2.0 Figure 3(a). Dual-luciferase reporter assay showed that miR-19b-3p overexpression dramatically decreased luciferase activity of TINCR-WT, while it had no effect on luciferase activity of TINCR-Mut (Figure 3(b), p < 0.05). RIP assay showed that TINCR directly targeted miR-19b-3p (Figure 3(c), p < 0.05). RT-qPCR assay showed that miR-19b-3p level was higher in DDP-sensitive tissues than in DDP-resistant tissues (Figure 3(d), p < 0.05), and miR-19b-3p level was higher in OCM-1/DDP cells than in OCM-1 cells (Figure 3(e), p < 0.05). In addition, TINCR knockdown increased miR-19b-3p level in OCM-1/DDP cells, while TINCR overexpression reduced miR-19b-3p level (Figure 3(f), p < 0.05). In CM tissues, TINCR level was negatively correlated with miR-19b-3p level (Figure 3(c), r = 0.624).

### MiR-19b-3p directly bound to ERK-2 in DDP-resistant CM cells

**Figure 4.**
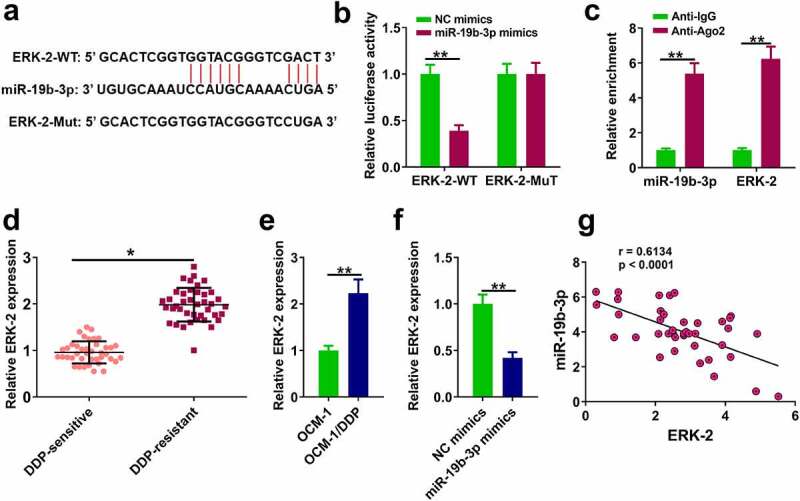
ERK-2 is a target of miR-19b-3p (a) The binding of miR-19b-3p to ERK-2 predicted by TargetScan software. (b) Luciferase reporter assay. (c) RIP assay was used to detect the correlations between ERK-2 and miR-19b-3p. (d) ERK-2 expression in DDP-resistant or DDP-sensitive CM tissues. (e) ERK-2 expression in DDP-resistant or DDP-sensitive CM cells. (f) ERK-2 expression in OCM-1/DDP cells was detected by RT-qPCR. (g) ERK-2 and miR-19b-3p expression levels were analyzed by Pearson analysis. **P < 0.01, *P < 0.05; n = 3.

The downstream target gene of miR-19b-3p was explored. ERK-2 was predicted to be a potential target of miR-19b-3p by TargetScan ver. 7.2 Figure 4(a). Dual-luciferase reporter assay showed that miR-19b-3p mimics reduced luciferase activity of ERK-2-WT, while it had no effect on luciferase activity of ERK-2-Mut (Figure 4(b), p < 0.01). RIP assay showed a direct interaction between miR-19b-3p and ERK-2 (Figure 4(c), p < 0.001). RT-qPCR assay showed ERK-2 level was remarkably higher in DDP-resistant tissues and OCM-1/DDP cells compared to their counterparts (Figure 4(d,e), p < 0.01), and miR-19b-3p mimics inhibited ERK-2 expression (Figure 4(f), p < 0.01). Pearson’s correlation analysis indicated there was an obviously negative correlation between ERK-2 and miR-19b-3p expression in CM tissues (p < 0.05, Figure 4g).

### MiR-19b-3p decreased the DDP resistance of CM cells by regulating ERK-2

**Figure 5.**
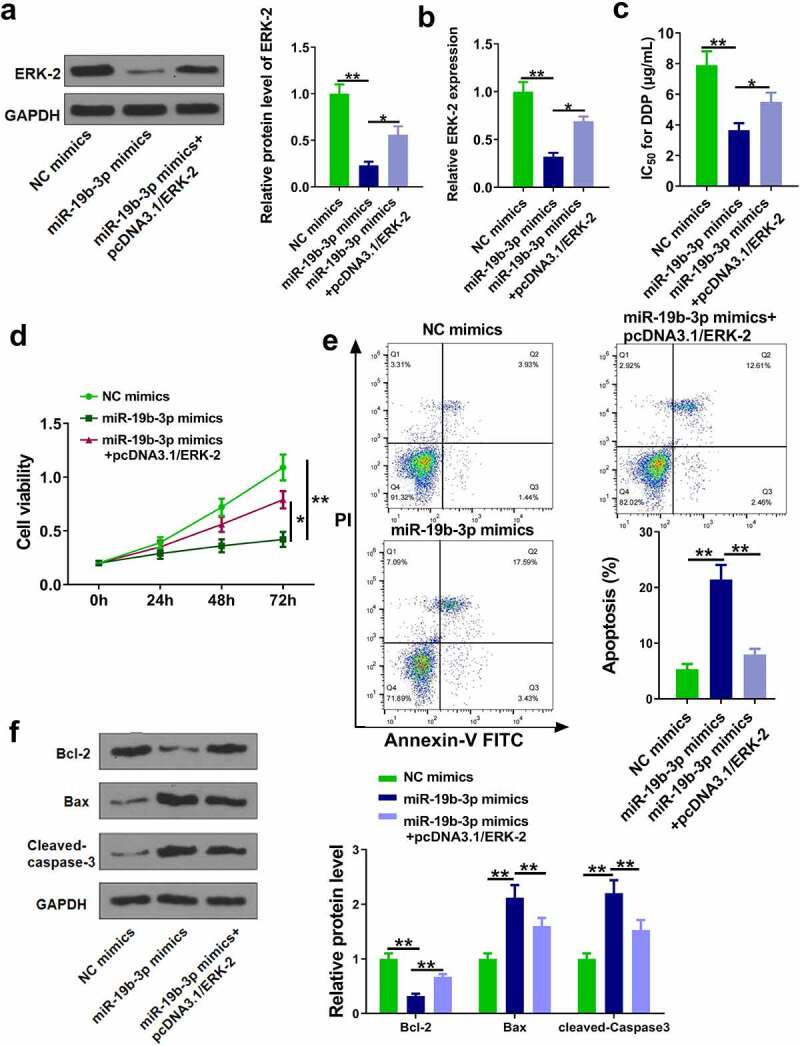
MiR-19b-3p reduces DDP resistance of CM cells. (a, b) ERK-2 expression was measured by Western blot and RT-qPCR. (c) IC_50_ values in OCM-1/DDP cells after transfection was measured. (d, e) OCM-1/DDP cell viability and apoptosis. (e, f) Bcl-2, Bax, and cleaved-caspase-3 expression. **P < 0.01, *P < 0.05; n = 3.

The role of ERK-2 in miR-19b-3p-mediated DDP resistance was investigated. OCM-1/DDP cells were transfected with miR-19b-3p mimics and pcDNA3.1/ERK-2. RT-qPCR and Western blot experiments showed that miR-19b-3p mimics markedly reduced ERK-2 level, while its effect was attenuated by pcDNA3.1/ERK-2 (Figure 5(a,b), p < 0.05). As shown in Figure 5(c), miR-19b-3p mimics significantly reduced IC_50_ values, while its effect was neutralized by pcDNA3.1/ERK-2 (p < 0.05). CCK-8 and flow cytometry assays showed that miR-19b-3p mimics inhibited cell proliferation and promoted cell apoptosis, while its effects were abolished by pcDNA3.1/ERK-2 (Figure 5(d,e), p < 0.05). Moreover, miR-19b-3p mimics reduced Bcl-2 and increased cleaved-caspase-3 and Bax, while its effects were attenuated by pcDNA3.1/ERK-2 (Figure 5(f), p < 0.05). These data suggested that miR-19b-3p reduced DDP resistance in CM cells by regulating ERK-2.

### TINCR regulated DDP resistance in CM via regulating miR-19b-3p/ERK-2 axis

**Figure 6.**
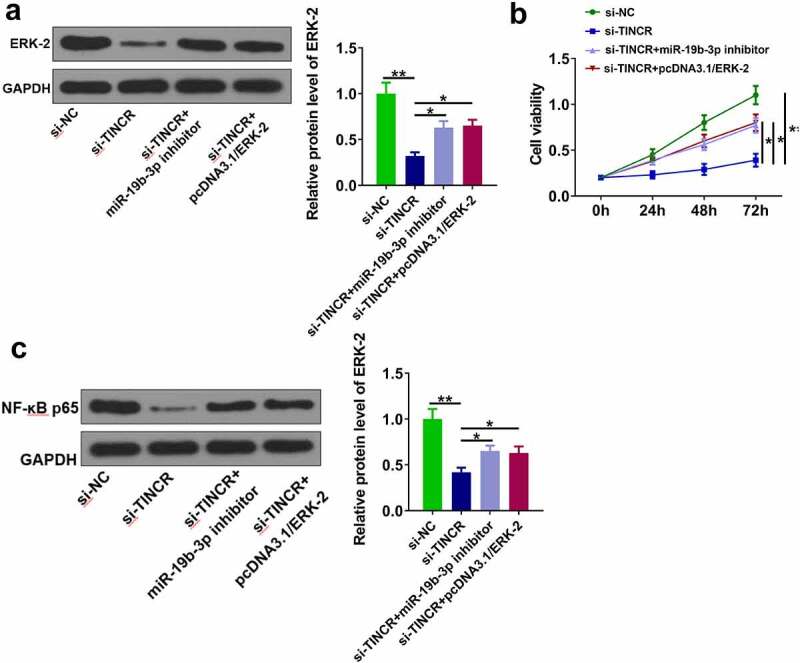
TINCR/miR-19b-3p/ERK-2 axis in CM cells. (a) Western blot analysis was used to measure ERK-2 protein expression. (b) IC_50_ values and cell viability. (c) NF-κB p65 protein expression. **P < 0.01, *P < 0.05; n = 3.

Finally, we investigated whether TINCR functioned in CM by regulating miR-19b-3p/ERK-2 axis. OCM-1/DDP cells were transfected with si-TINCR, miR-19b-3p inhibitor, and pcDNA3.1/ERK-2. The results showed that si-TINCR significantly reduced ERK-2 level, while its effect was abolished by miR-19b-3p inhibitor or pcDNA3.1/ERK-2 (Figure 6(a), p < 0.05). CCK-8 assay showed that si-TINCR obviously inhibited cell proliferation, while its effect was attenuated by miR-19b-3p inhibitor or pcDNA3.1/ERK-2 (Figure 6(b), p < 0.05). Moreover, si-TINCR significantly reduced NF-κB p65 expression, while its effect was alleviated by miR-19b-3p inhibitor or pcDNA3.1/ERK-2 (Figure 6(c), p < 0.05). These data suggested that TINCR enhanced DDP resistance of CM cells by regulating miR-19b-3p/ERK-2 axis.

## Discussion

CM is the most common malignant tumor in adults, and the clinical treatment of CM is mainly by surgery and radiotherapy along with chemotherapy [31,32]. Although cisplatin is widely used in CM treatment [33], the clinical efficacy of cisplatin is limited because of drug resistance. Therefore, resolving tumor chemotherapy resistance is extremely valuable for CM treatment. In this study, TINCR was found to be upregulated in CM, especially in DDP resistant CM. MiR-19b-3p was targeted by TINCR. ERK-2 was a target of miR-19b-3p. These results suggested that TINCR regulated ERK-2 expression and increased DDP resistance of CM cells through miR-19b-3p.

Although lncRNAs do not encode proteins and are transcripts similar to mRNAs, they are key regulators of the development of various cancers. For example, lncRNA HOTAIR downregulation enhances cervical cancer sensitivity to radiotherapy [34]. LncRNA NCK1-AS1 inhibition enhances cervical cancer cell sensitivity to cisplatin [35]. LncRNA SNHG3 promotes epithelial-stromal transformation, enhances sorafenib resistance, and reduces the survival rate of patients with liver cancer [36]. Therefore, studies on the role and potential mechanism of lncRNAs in radiosensitivity and chemosensitivity are important. Previous studies have revealed that TINCR plays an important role in multiple cancers. For example, TINCR can promote hepatocellular carcinoma proliferation and invasion via regulating STAT3 signaling [37]. However, the role of TINCR in CM remains unknown. Here, we revealed that TINCR is abnormally upregulated in DDP-resistant CM cells. TINCR overexpression enhances the DDP resistance of CM cells while its knockdown reduces the DDP resistance of CM cells, suggesting that TINCR plays a positive role in the production of DDP resistance in CM cells. Moreover, we also detected the effects of TINCR on cell proliferation and apoptosis. We found that TINCR overexpression promotes CM cell proliferation and inhibits CM cell apoptosis, while its knockdown exhibits opposite effects. Therefore, TINCR upregulation may promote tumorigenesis. To our knowledge, our study is the first to report the oncogenic role of TINCR in CM.

LncRNA can target mRNA to promote cell autophagy [38]. It has been found that some miRNAs are related to chemosensitivity and drug resistance. For example, miR-16 and miR-15b expression are reduced in SGC7901/VCR cell line [39], and overexpression of miR-16 or miR-15b enhances anti-tumor drug sensitivity in SGC7901/VCR cell lines. In addition, miR-15b overexpression can enhance the chemosensitivity of SGC7901/VCR cell line [40]. MiR-19b-3p could induce gastric cancer apoptosis [41]. Here, as a target gene of TINCR, miR-19b-3p in DDP-tolerated CM tissues and cell lines is reduced. Moreover, Pearson’s correlation analysis revealed an obviously negative correlation between TINCR and miR-19b-3p expression in CM tissues.

As a MAPK signal transduction pathway [42], ERK pathway is related to many biological behaviors, such as cell growth, transformation, and drug sensitivity. In this study, ERK-2 is a target gene of miR-19b-3p, and its expression is enhanced in DDP-tolerated CM cell lines and tissues. ERK-2 is a key regulator in DDP resistance of CM. Moreover, ERK-2 and NF-κB p65 expression levels are reduced by TINCR and these reductions are abolished by miR-19b-3p inhibitor and ERK-2 overexpression. The present study is the first to suggest that the TINCR/miR-19b-3p/ERK-2/NF-κB p65 axis is involved in DDP resistance of CM.

Increasing studies have focused on the mechanism of drug resistance in CM. A recent study has revealed that circRNA circBIRC6 could regulate DDP resistance of CM by regulating miR-503-3p/ERK axis [26]. Here, our study also revealed that lncRNA TINCR can enhance DDP resistance in CM via modulating miR-19b-3p/ERK-2 axis, underling that ERK signaling is an important downstream pathway in the regulatory mechanisms of DDP resistance in CM. However, other signaling pathways have also been reported to be involved in the molecular mechanisms of DDP resistance. Therefore, our future studies will continue to explore more factors involved in DDP transport pathways.

Our study also has limitations. First, the sample size of our collected tissues from CM patients is not big enough. Our future study will collect more samples to further support our findings. In addition, our results also need to be verified by animal experiments.

## Conclusion

TINCR regulates CM cell DDP resistance by modulating ERK-2 and NF-κB expression via sponging miR-19b-3p, suggesting that TINCR may be a treatment target for DDP-resistance CM patients.

## Supplementary Material

Supplemental Material

## Data Availability

The datasets used and/or analyzed during the current study are available from the corresponding author on reasonable request.
