# Supplementary material for: Long non-coding RNA terminal differentiation-induced non-coding RNA regulates cisplatin resistance of choroidal melanoma by positively modulating extracellular signal-regulated kinase 2 via sponging microRNA-19b-3p
Source: Bioengineered. 2022 Jan 22;13(2):3422–33. doi: 10.1080/21655979.2021.2014618 (PMC8973966; doi:10.1080/21655979.2021.2014618)

**Supplementary_Material 1** The origianl Western blot images whose histogram has been shown in Figure 2E


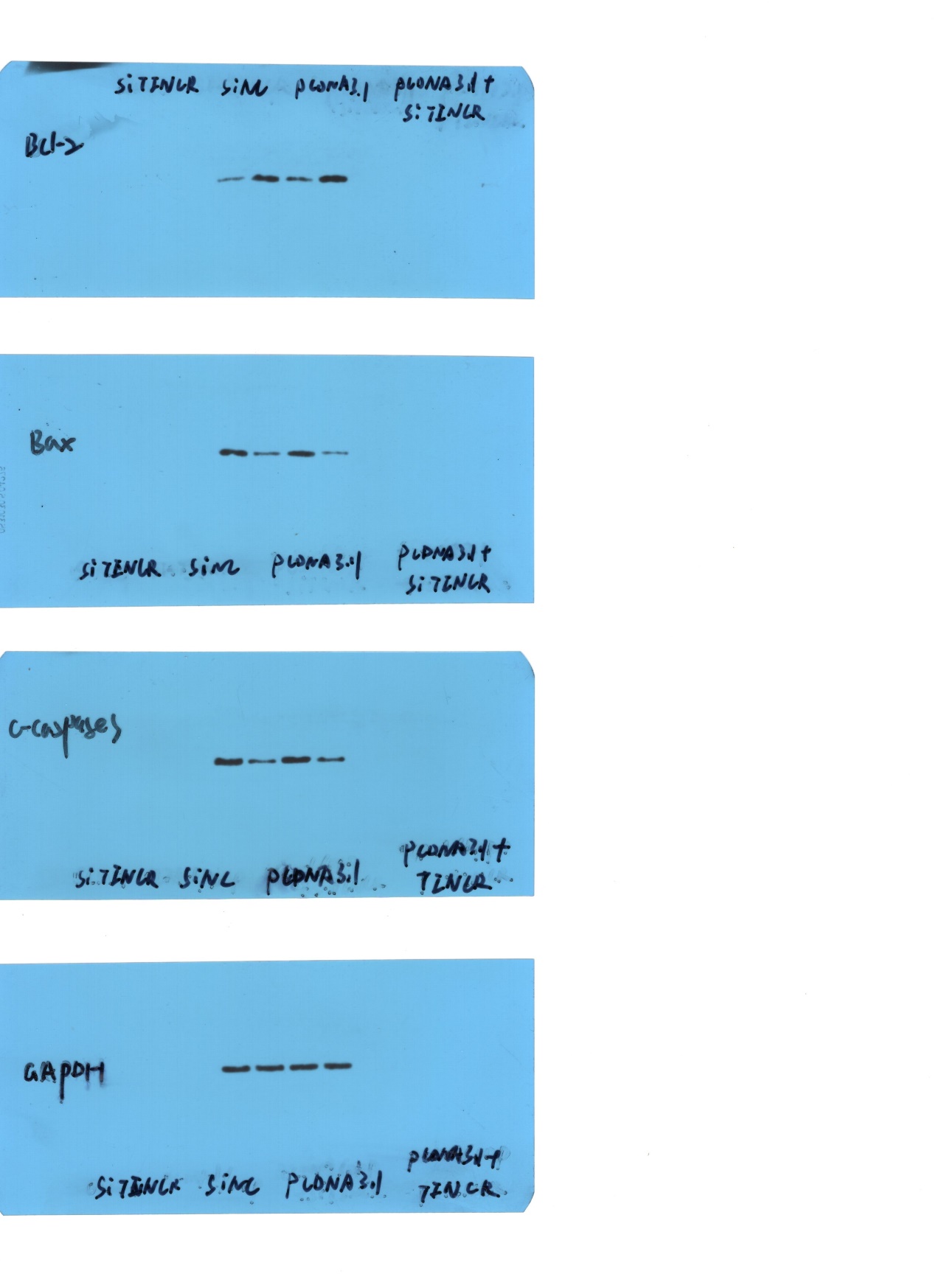


**Supplementary_Material 2.** The origianl western blot images whose histogram has been shown in Figure 5A

**
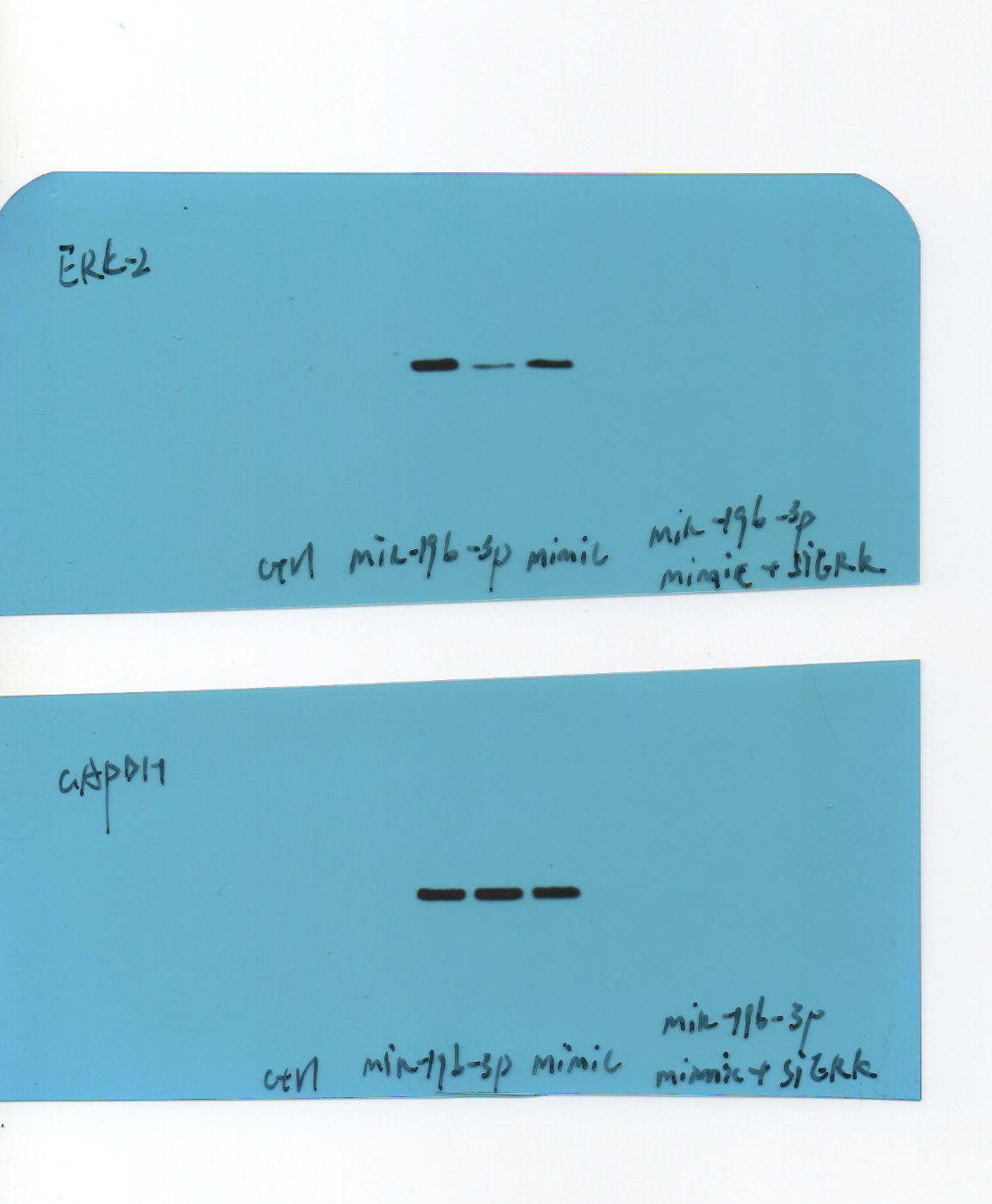
**

**Supplementary_Material 3.** The origianl western blot images whose histogram has been shown in Figure 5F


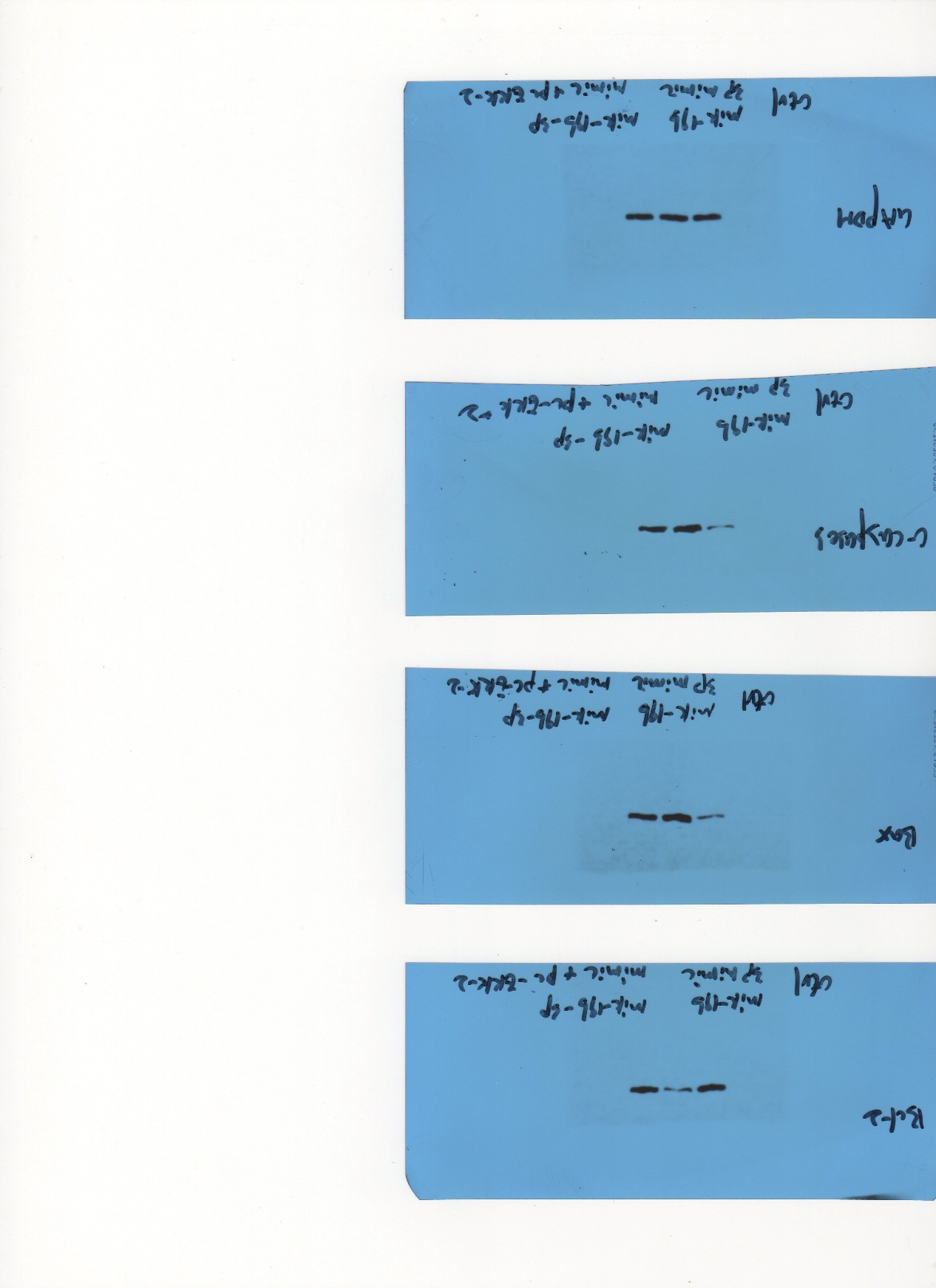


**Supplementary_Material 4.** The origianl western blot images whose histogram has been shown in Figure 6A


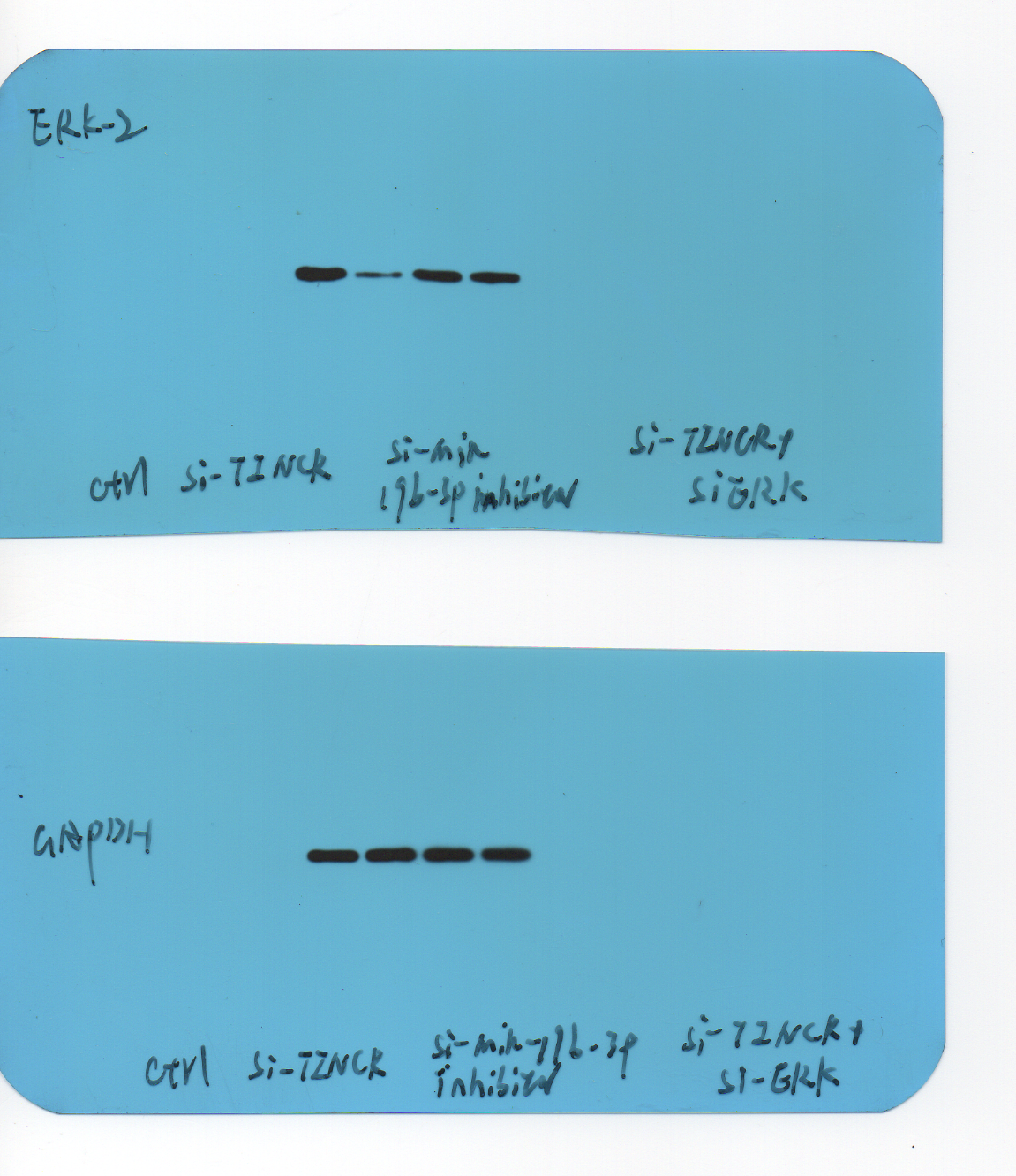


**Supplementary_Material 5.** The origianl western blot images whose histogram has been shown in Figure 6C


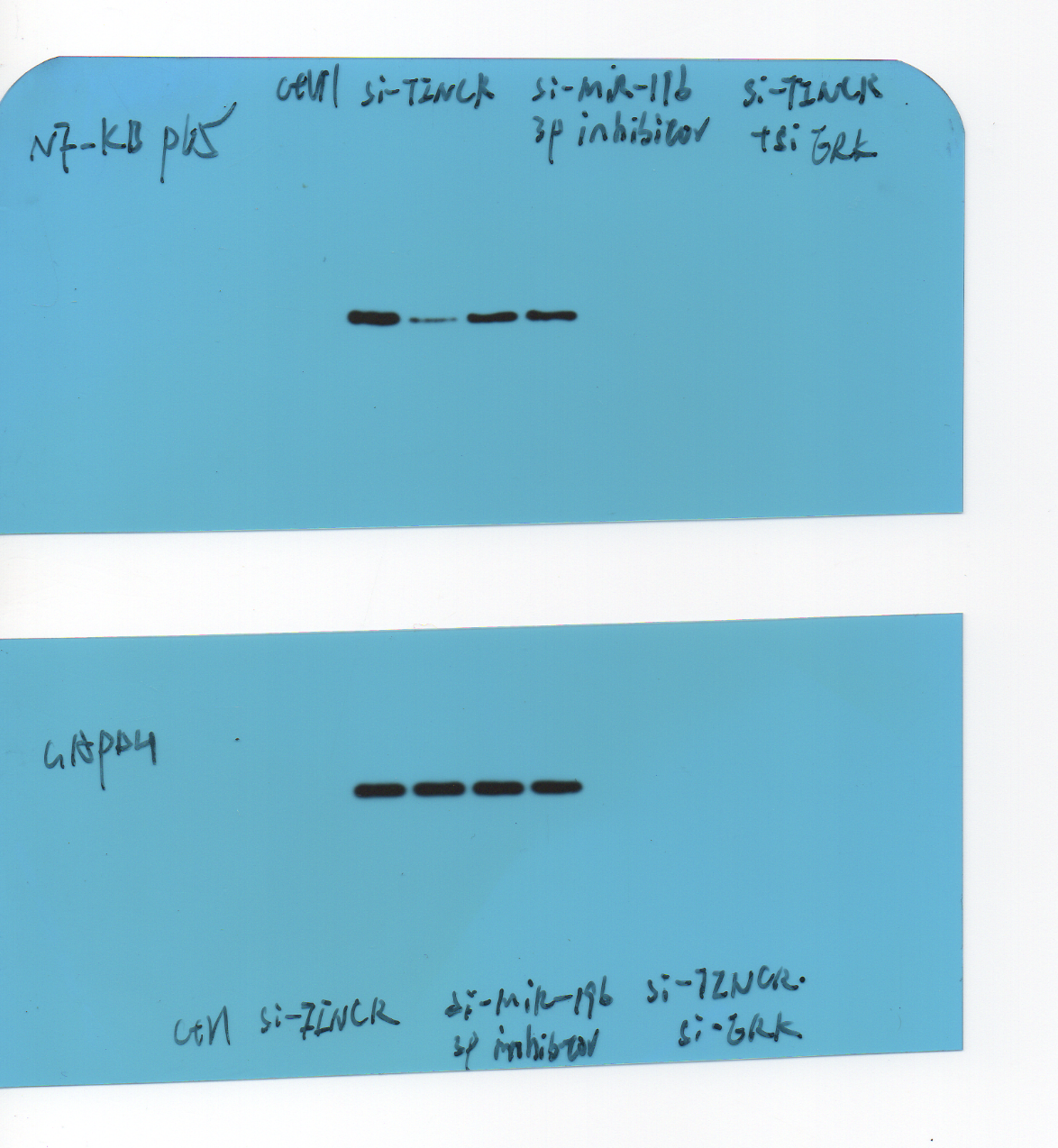

Supplement: Supplemental Material [file KBIE_A_2014618_SM6175.zip › supplementary/Supplementary_Materials.docx]
